# High density of TCF1+ stem-like tumor-infiltrating lymphocytes is associated with favorable disease-specific survival in NSCLC

**DOI:** 10.3389/fimmu.2024.1504220

**Published:** 2024-12-19

**Authors:** Dagny Førde, Thomas Kilvær, Mona Irene Pedersen, Egil S Blix, Ilona Urbarova, Erna-Elise Paulsen, Mehrdad Rakaee, Lill-Tove Rasmussen Busund, Tom Donnem, Sigve Andersen

**Affiliations:** ^1^ Department of Clinical Medicine, UiT The Arctic University of Norway, Tromsø, Norway; ^2^ Department of Oncology, University Hospital of North Norway, Tromsø, Norway; ^3^ Department of Community Medicine, UiT The Arctic University of Norway, Tromsø, Norway; ^4^ Department of Pulmonology, University Hospital of North Norway, Tromsø, Norway; ^5^ Department of Medical Biology, UiT The Arctic University of Norway, Tromsø, Norway; ^6^ Department of Clinical Pathology, University Hospital of North Norway, Tromsø, Norway; ^7^ Department of Cancer Genetics, Oslo University Hospital, Oslo, Norway

**Keywords:** NSCLC, digital pathology, CD8, PD1, TCF1, machine learning

## Abstract

**Introduction:**

Tumor-infiltrating lymphocytes are both prognostic and predictive biomarkers for immunotherapy response. However, less is known about the survival benefits oftheir subpopulations.

**Methods:**

Using machine learning models, we assessed the clinical association of the CD8+, PD1+, TCF1+ cel l subset by multiplex immunohistochemistry using tissue microarrays in 553 non-small cell lung cancer (NSCLC) patients and its correlation with other immune cell biomarkers.

**Results:**

We observed positive correlations between TCF1 and CD20 (r=0.37), CD3 (r=0.45)and CD4 (r=0.33). Notably, triple positive (CD8+PD1+TCF1+) were rare, only observed in 29 of 553 patients (5%). Our analysis revealed that cells coexpressing TCF1 with either CD8+ or PD1+ were independent prognostic markers of disease-specific survival in multivariable analysis (HR=0.728, p=0.029 for CD8+TCF1+, and HR=0.612, p=0.002 for PD1+TCF1+). To pilot the subtype of abundant CD8-TCF1+ cells, we explored an immune cell infiltrated whole slideimage and found the majority to be CD4+.

**Discussion:**

Overall, these findings suggest that assessment of CD8+, PD1+, TCF1+ could serve as a potential prognostic biomarker in NSCLC.

## Introduction

Lung cancer remains the leading cause of cancer-related deaths, and globally it accounts for approximately 1.8 million fatalities every year (1). In Norway, lung cancer constitutes 10% of all new cancer diagnoses (2, 3). Lung cancer is divided into two main groups: non-small cell lung cancer (NSCLC) and small cell lung cancer (SCLC), of which the former accounts for approximately 85%. NSCLC is a heterogeneous disease, comprising various histopathological subtypes, with adenocarcinoma being the most prevalent (4).

The immune system plays a critical role in combating cancer development and progression. This process is tightly regulated by both activating and inhibitory signals (5). Consequently, the ability to evade immune detection and response is recognized as a hallmark of cancer (6). CD8+ cytotoxic T lymphocytes (CTLs) are the most powerful effectors in the anticancer immune response and are regarded as the main immune cells targeting established and developing tumors (7), through elimination of cancer cells by release of cytotoxic granules and induction of apoptosis (8). Immune checkpoint proteins (ICPs), such as programmed death-1 receptor (PD1), programmed death-ligand 1 (PD-L1) and CTL-associated antigen 4 (CTLA-4), play a crucial role in regulating self-tolerance under normal conditions. However, upregulation of ICPs during cancer development causes suppression of the immune system, leading to a less efficient anti-tumor response (8).

Important subtypes of CTLs are being investigated, including diverse types of effectors, hyporesponsive and dysfunctional cells (9, 10). A key subtype of CTLs known as exhausted T-cells (Tex), represents CTLs that lose their ability to eliminate cancer cells due to antigen persistence (11). Tex was initially observed in chronic infections and characterized by expression of the PD1 inhibitory receptor. More recently, a subtype of CTLs expressing both PD1 and T-cell factor 1 (TCF1) (CD8+PD1+TCF1+) has been proposed as progenitor cell for Tex (Tpex) (12, 13). The transcription factor TCF1, encoded by the human *tcf7* gene, is an important regulator of T-cell development and plays a key role in differentiation of memory CD8+ T cells in acute and chronic infections and in cancer (14). TCF1 and its homologue LEF1 are expressed in multiple isoforms in T-cells, functioning as transcription factors largely independently of classical Wnt signaling pathway (15). In healthy individuals, high expression of both TCF1 and LEF1 specifically marks memory CD8+ T cells with capacity for self-renewal, maintenance of high T-cell receptor (TCR) clonal diversity and show stronger proliferative response to TCR stimulation (16). Notably, in tumor tissue TCF1 is predominantly expressed in T-cells located in tertiary lymphoid structures (TLSs) and not in tumor parenchyma (17, 18), where Tex is more abundant. In addition, TCF1 is currently being investigated as a potential clinical biomarker for assessing the efficacy of immunotherapy or viral control in HIV and hepatitis C treatment (16, 19), suggesting TCF1 as potential clinical biomarker in the future.

Double positive CD8+TCF1+ cells exhibit functional traits of both memory and of exhaustion, retaining self-renewal and differentiation, typical for memory cells, while also showing qualities of exhaustion that limit their effector functions (13). CTLs cells that undergo proliferation after PD1 blockade exhibit stem-cell features like self-renewing and TCF1 is essential in forming this subtype (20). The presence of CD8+TCF1+ cells has been identified as a potential predictor of favorable response to immune check point inhibitors (ICIs) in NSCLC patients (21–23). Previous studies on TCF1 in NSCLC have primarily focused on the predictive potential of CD8+PD1+TCF1+ cells in blood and tissue of small patient cohorts receiving ICIs. Data regarding the infiltration of lymphocytes exhibiting the different combinations of these markers in NSCLC are limited. To address this, we investigated their presence and prognostic impact in a large, unselected cohort of treatment-naive resected NSCLC patients using machine learning to detect multiplex-stained subsets of CD8+, PD1+, TCF1+ cells in TMA cores.

## Materials and methods

### Patient population

The initial study population comprised 633 stage I to III NSCLC patients who underwent radical resection at the University Hospital of North Norway or Nordland Central hospital between 1990-2010. Following exclusion after predetermined criteria (radiotherapy or chemotherapy prior to surgery, other malignancy within 5 years prior to NSCLC diagnosis, inadequate formalin-fixed paraffin-embedded (FFPE) blocks), 553 patients were eligible for further analyses. The median follow-up of survivors was 86 months (about 7 years), with the last update in October 2013. The patient cohort has been thoroughly documented in prior publications (24, 25).

### Tissue samples and tissue micro array (TMA) construction

The tissue sampling procedure for this cohort has been extensively documented (26, 27). Briefly, tissue specimens were retrieved from the archives at the participating hospitals. Experienced pathologists reviewed each sample and marked areas representing tumor and stroma on the overview hematoxylin and eosin (H&E) stained slides. For each patient, duplicate 0.6mm cores for both tumor and stroma were collected and transferred to a recipient TMA block. Eleven blocks comprising primary tumor and tumor associated stroma were made to accommodate the entire cohort. For this study, one 4µm thick tissue slide was cut from each block using a microtome (Microm microtome HM355S) and used for subsequent analyses. One whole tissue FFPE slide from a patient with dense immune infiltration was used for a pilot multiplex CD4+, PD1+, TCF1+ cell subset analysis.

### Immunohistochemistry (IHC)

IHC triple staining was performed using Discovery Ultra Research instrument Roche 05987750001. Validated antibodies for immunohistochemistry (IHC-P) were sourced from Cell Signaling, and antibodies approved for *in vitro* diagnostic (IVD) use from Ventana Roche/Sigma Aldrich. Each antibody was optimized as a single stain in-house before using together in a multiplex assay. The order of antibodies and chromogens for triple staining was tested to ensure optimal antigen localization and chromogen expression. Controls included whole lung cancer tissue and a multi-tissue TMA control of positive and negative tissues, used throughout optimization and final runs. Detailed protocols for the optimized IHC processes are provided in **Supplementary Tables S1** (for CD8, PD1 and TCF1) and S2 (for CD8, CD4 and TCF1), with product information for antibodies and reagents listed in **Supplementary Table S3**.

### Scoring of TMA-cores

After staining, TMA slides were digitized using a Pannoramic 250 Flash III slide scanner (3DHistech, Budapest, Hungary), and processed using QuPath version 0.5.1 (28). First, TMA cores were assigned to their corresponding patient ID. Prior to analysis, preprocessing was conducted to calculate color features. Cores containing less than 40% of the expected tissue area were automatically excluded using a script. The remaining cores were manually curated under supervision of an experienced pathologist (LTB) and cores with obvious damage, poor staining, low tissue quality, predominant necrosis or normal lung tissue and insufficient number of tumor cells identified, were excluded.

Cell detection was performed using the deep-learning algorithm “StarDist” within QuPath (29). To classify cells into all eight combinations of CD8+, PD1+ and TCF1+, a random forest classifier was trained on manually annotated cells from the TMAs. After classifications, the mean number of cells per mm^2^ of tissue across tissue cores was calculated for each class and exported to R and SPSS. In survival analyses, high and low density was determined using a median cut-off approach for widely occurring cell types and an “any/none” approach for scarce cell types.

### Statistical methods

All statistical analyses were conducted in RStudio 2023.12.1 Build 402 and R version 4.3.1 using the packages “*cowplot”, “ggplot2”, “gtable”, “grid”, “gridExtra, “Hmisc*” and “*reshape2*” or in SPSS 29.0 (Chicago, IL). Associations between clinicopathological variables and dichotomized cell types were investigated using χ2 and Fisher’s exact tests, whenever appropriate. Correlations between cell type densities investigated in this study, and immune markers from previous studies using the same cohort, were investigated using Spearman correlations coefficients. Results with p-values <0.05 were highlighted. For survival analyses, disease-specific survival (DSS) was defined as the time between reception of the surgical specimen at the Pathology department and lung cancer death. Univariable survival analyses of different combinations of CD8+, PD1+ and TCF1+ cells, as well as clinicopathological variables, were visualized using Kaplan-Meier curves and their statistical difference determined by the log-rank test. For multivariable survival analyses, backward conditional Cox-regression was performed with probabilities for stepwise entry and removal set to 0.05 and 0.10, respectively. Clinicopathological variables with p-values <0.05 from the univariable analyses were entered into the multivariable analyses.

## Results

### Patient characteristics

This work used tissue samples, clinicopathological variables and follow-up data from 553 NSCLC patients in stage I-III (**Tables 1**, **2**). Age at diagnosis ranged between 28-85 years with a median age of 67. Most patients were males (68%) and only 4% stated that they were never-smokers.

**Table 1:** Clinicopathological variables and investigated markers in our cohort of NSCLC patients.

	A)					B)								
						CD8			PD1			TCF1		
	N (%)	5Y	Median	HR (95% CI)	P	Low	High	P	Low	High	P	Low	High	P
Age					0.643			0.654			0.005			0.654
<65	231(42)	58	127	1		105	111		124	92		105	111	
≥65	322(58)	59	NR	0.94(0.72-1.22)		149	143		130	162		149	143	
Gender					0.025			0.395			0.508			<0.001
Female	180(33)	64	190	1		78	88		79	87		61	105	
Male	373(67)	55	91	1.39(1.06-1.83)		176	166		175	167		193	149	
Weight loss					0.958			0.893			0.185			0.448
<10%	497(90)	58	190	1		228	229		224	233		232	225	
>10%	55(10)	59	NR	1.01(0.64-1.61)		26	24		30	20		22	28	
Missing	1(0)													
Smoking					0.069			0.283			0.630			0.412
Never smoked	21(4)	50	105	1		12	6		11	7		11	7	
Present smoker	350(63)	62	235	0.66(0.32-1.33)		157	168		161	164		166	159	
Previous smoker	182(33)	52	84	0.88(0.42-1.82)		85	80		82	83		77	88	
ECOG					0.009			0.523			0.611			0.165
0	324(59)	63	235	1		143	155		144	154		141	157	
1	191(35)	52	71	1.51(1.14-2)		91	83		90	84		97	77	
2	38(7)	52	NR	1.46(0.78-2.72)		20	16		20	16		16	20	
Histology					0.095			0.698			0.067			0.004
LUSC	307(56)	64	235	1		145	138		153	130		159	124	
LUAD	239(43)	52	73	1.25(0.96-1.63)		107	111		99	119		93	125	
Missing	7(1)													
tStage					<0.001			0.413			0.606			0.164
T1	180(33)	72	235	1		88	78		80	86		72	94	
T2	208(38)	54	83	1.87(1.38-2.53)		89	100		91	98		97	92	
T3	104(19)	56	NR	1.69(1.16-2.46)		44	51		50	45		54	41	
T4	61(11)	31	21	3.44(2.04-5.79)		33	25		33	25		31	27	
nStage					<0.001			0.746			0.980			0.012
N0	379(69)	69	235	1		176	174		174	176		160	190	
N1	118(21)	36	35	2.81(1.96-4.03)		55	52		54	53		66	41	
N2	56(10)	22	21	4.13(2.4-7.11)		23	28		26	25		28	23	
pStage					<0.001			0.676			0.829			0.016
I	232(42)	74	235	1		110	102		103	109		90	122	
II	185(33)	59	114	1.7(1.27-2.28)		81	90		86	85		95	76	
III	136(25)	28	21	4.04(2.78-5.87)		63	62		65	60		69	56	
Differentiation					<0.001			0.124			0.083			0.747
Poor	232(42)	48	51	1		96	116		94	118		104	108	
Moderate	240(43)	63	190	0.66(0.5-0.89)		116	108		119	105		116	108	
Well	81(15)	73	NR	0.39(0.27-0.58)		42	30		41	31		34	38	
Vascular Infiltration					<0.001			0.891			0.286			0.575
No	453(82)	62	235	1		206	209		213	202		205	210	
Yes	97(18)	38	35	1.93(1.31-2.84)		46	44		40	50		48	42	
Missing	3(1)													
CD8 total					0.001									
Low	254(46)	52	71	1										
High	254(46)	65	235	0.63(0.48-0.83)										
Missing	45(8)													
PD1 total					0.025									
Low	254(46)	55	91	1										
High	254(46)	62	235	0.73(0.55-0.96)										
Missing	45(8)													
TCF1 total					0.002									
Low	254(46)	53	84	1										
High	254(46)	64	NR	0.64(0.49-0.85)										
Missing	45(8)													

5Y, Five-year disease-specific survival; Median, Median survival; HR, Hazard ratio; ECOG, Eastern Cooperative Oncology Croup Performance Status Scale; T-stage, Tumor stage; N-stage, Node stage; P-stage, Pathological stage; LUSC, Lung squamous cell carcinoma; LUAD, Lung adenocarcinoma; NR, Not reached.

**A)** Clinicopathological variables and investigated markers as predictors of disease-specific survival in NSCLC patients (n=553, univariable analyses, log-rank test), **B)** Investigated markers and their associations with clinicopathological variables (n=508, χ^2^ and Fisher’s exact tests as appropriate).

**Table 2:** Immune cell subgroups.

	N (%)	5Y	Median	HR (95%CI)	P
CD8+PD1+TCF1+					0.308
None	479(87)	58	190	1	
Any	29(5)	67	NR	0.69(0.38-1.26)	
Missing	45(8)				
CD8+PD1+TCF1-					0.844
Low	254(46)	60	190	1	
High	254(46)	57	235	1.03(0.78-1.35)	
Missing	45(8)				
CD8+PD1-TCF1+					0.003
Low	254(46)	53	83	1	
High	254(46)	64	NR	0.66(0.5-0.87)	
Missing	45(8)				
CD8+PD1-TCF1-					0.002
Low	254(46)	52	71	1	
High	254(46)	65	235	0.65(0.49-0.85)	
Missing	45(8)				
CD8-PD1+TCF1+					<0.001
None	324(59)	53	84	1	
Any	180(33)	67	235	0.59(0.45-0.79)	
Missing	49(9)				
CD8-PD1+TCF1-					0.054
Low	254(46)	56	104	1	
High	254(46)	61	235	0.76(0.58-1)	
Missing	45(8)				
CD8-PD1-TCF1+					0.004
Low	254(46)	53	98	1	
High	254(46)	64	NR	0.67(0.51-0.88)	
Missing	45(8)				

5Y, Five-year disease-specific survival; Median, Median survival; HR, Hazard ratio; NR, Not reached.

Immune cell subgroups as predictors for disease-specific survival in NSCLC patients (n=553, univariable analyses, log-rank test). Median density values or any/none were used as cut-off values.

### Immune cell distribution and their classification

**Figure 1:**
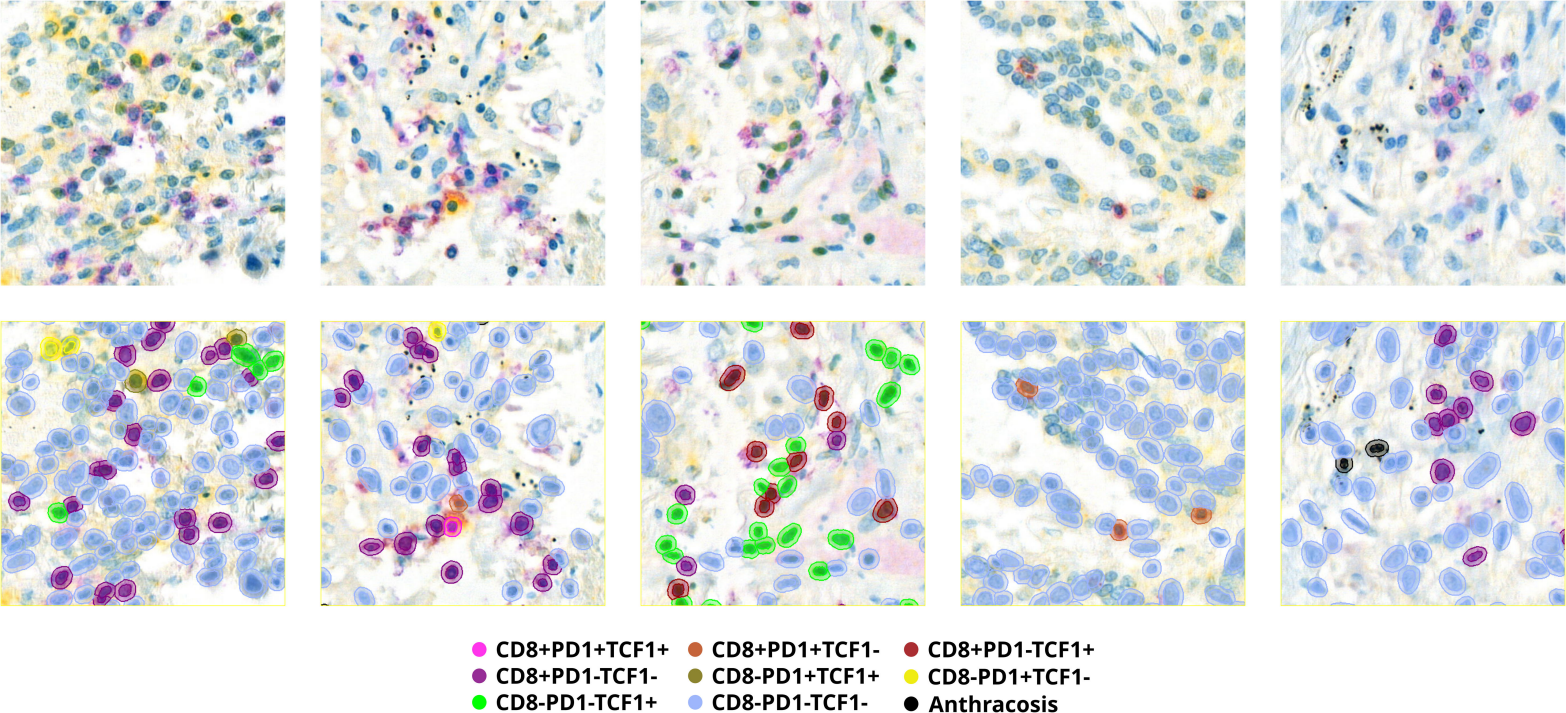
Five scanned images depicting immune cells stained by CD8, PD1 and TCF1 markers using IHC in the panel above with corresponding overlay obtained from QuPath classifier in the panel below, classifying cells into eight different combinations based on CD8+/-, PD1+/-and TCF1+/- expression or anthracosis.

The classifier in QuPath was trained with manual annotations on the TMA-slides. **Figure 1** provides a visual representation of the classifier and the colors corresponding to the eight distinct expression combinations. Among the stained cells, single CD8+ cells were most prevalent, followed by TCF1+ and lastly PD1+ cells, with mean density expressions of 294/mm^2^, 120/mm^2^ and 33/mm^2^, respectively. **Supplementary Figure S1** further illustrated distribution of expression densities for the different cell subtypes.

**Figure 2:**
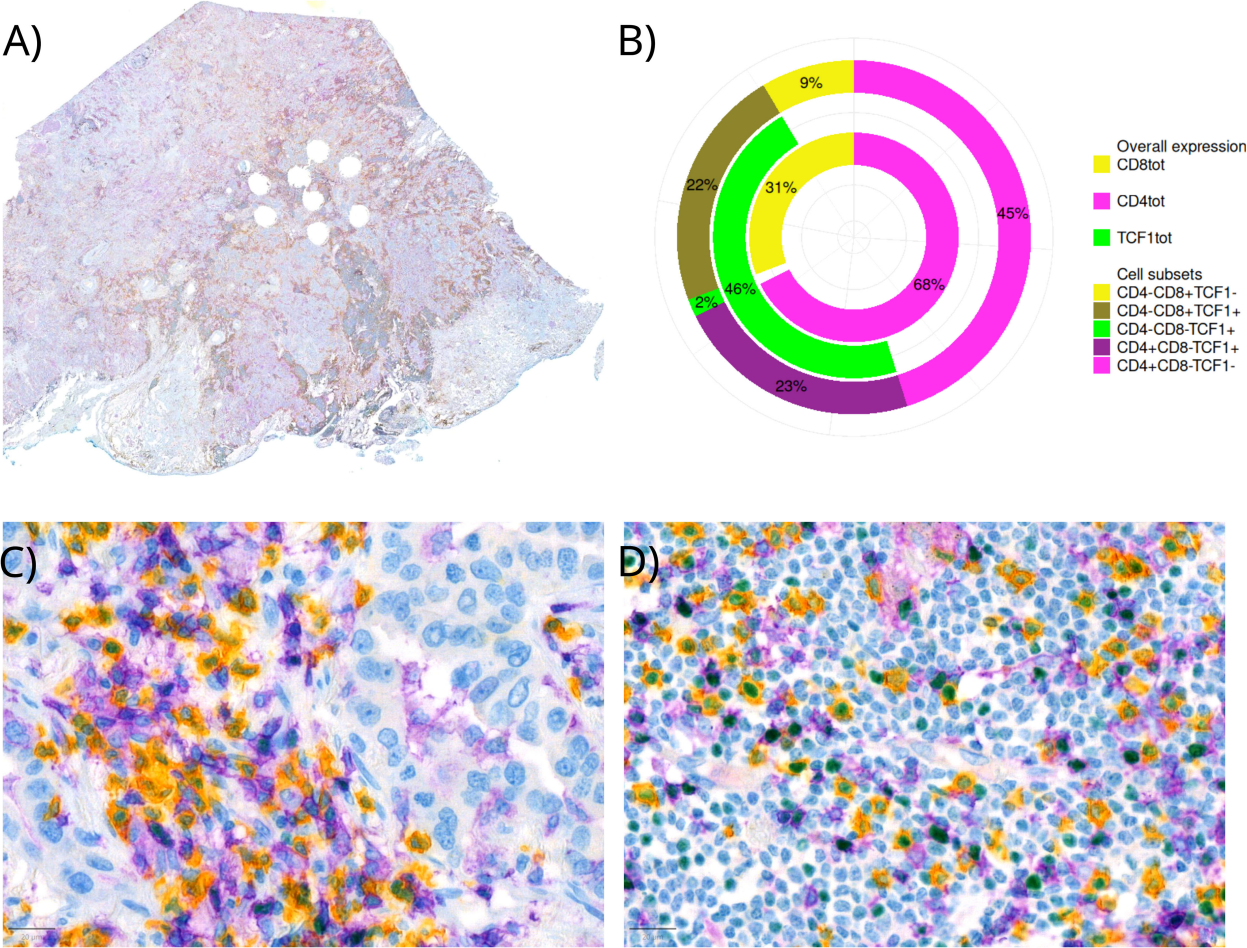
Triple IHC staining of CD8+/-, CD4+/- and TCF1+/- on a whole FFPE slide. Yellow staining represents CD8+, purple CD4+ and green TCF1+ cells. **(A)** A whole slide image **(B)** Percentages of lymphocyte subsets in the respective whole slide visualized by different colors for different surface (CD8+ and CD4+) and nuclear (TCF1+) markers **(C)** Immune cell infiltration in tumor **(D)** Immune cell infiltration in stroma.

Due to the high number of CD8-PD1+/-TCF1+cells and a moderate correlation between CD4+ and TCF1+ cells (section below), we aimed to identify these further. Morphologically, they looked like lymphocytes, and the hypothesis was that these were CD4+ T cells. Consequently, we identified a TMA core with an abundant lymphoid infiltration in our cohort and performed a triple IHC comprising of CD4+, CD8+, to identify lymphoid cells, and TCF1+ on one FFPE WSI. Using a similar QuPath pipeline as described above, we found that the majority of TCF1+ cells were either CD4+ (~2/3) or CD8+ (~1/3) and that single positive TCF1+ cells were rare. In addition, we observed a higher amount of CD8+ T cells in dense lymphoid aggregates, while CD4+ T cells were more prevalent in peripheral stroma. We found 68% of identified lymphoid cells to be CD4+, whereas 23% of these were TCF1+ (**Figure 2**). However, these findings should be interpreted with caution, as they are based only on a single patient slide.

### CD8+, PD1+, TCF1+ correlations with clinicopathological variables and other immune markers

**Figure 3:**
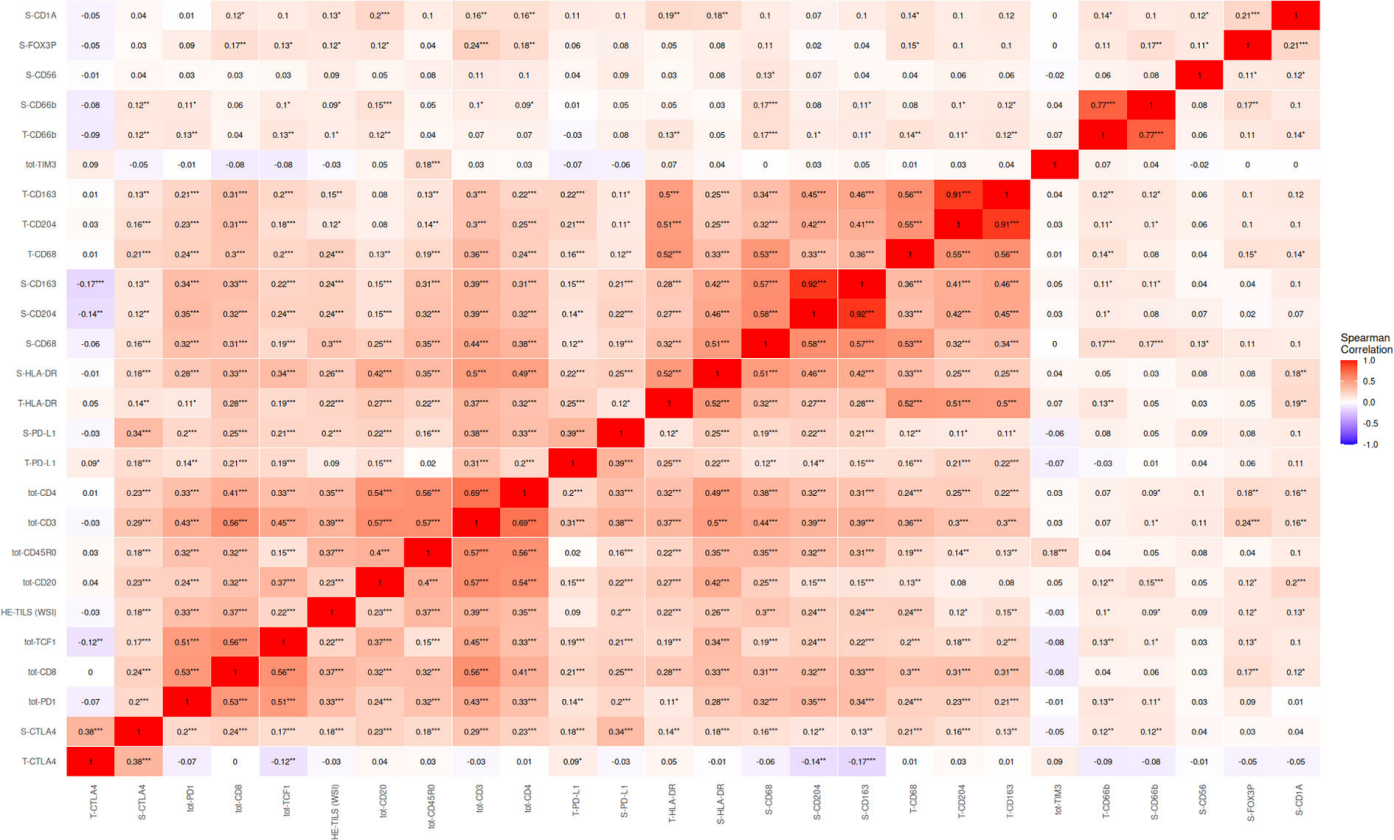
Correlations of immune markers in NSCLC. In this analysis, the total amount of CD8+, TCF1+ and PD1+ density is used. Spearman’s rank correlation coefficients range from -1 to 1, positive values implicate a direct relationship. Correlations are denoted * <0.05, ** <0.01, ***<0.001. Tot, total expression; S, Stromal expression; T, Tumor expression.

High density of TCF1 positive cells was associated with female gender, LUAD histology and early-stage disease. Correlations with other immune markers were assessed using Spearman’s rank correlation coefficients. Further, TCF1 showed a positive correlation with CD20 (r=0.37, p<0.001), CD3 (r=0.45, p<0.001) and CD4 (r=0.33, p<0.001). CD20 is a surface marker on mature B-cells, while CD3 is present on the surface of all T-cells. CD4 is mainly present on the surface of T helper cells, important for assisting other immune cells in recognizing and responding to pathogens. Additionally, we observed a correlation of TCF1 with HLA-DR (r=0.34, p<0.001) found on antigen presenting cells and which serves as a ligand for the T-cell receptor (**Figure 3**).

### Survival analysis

**Figure 4:**
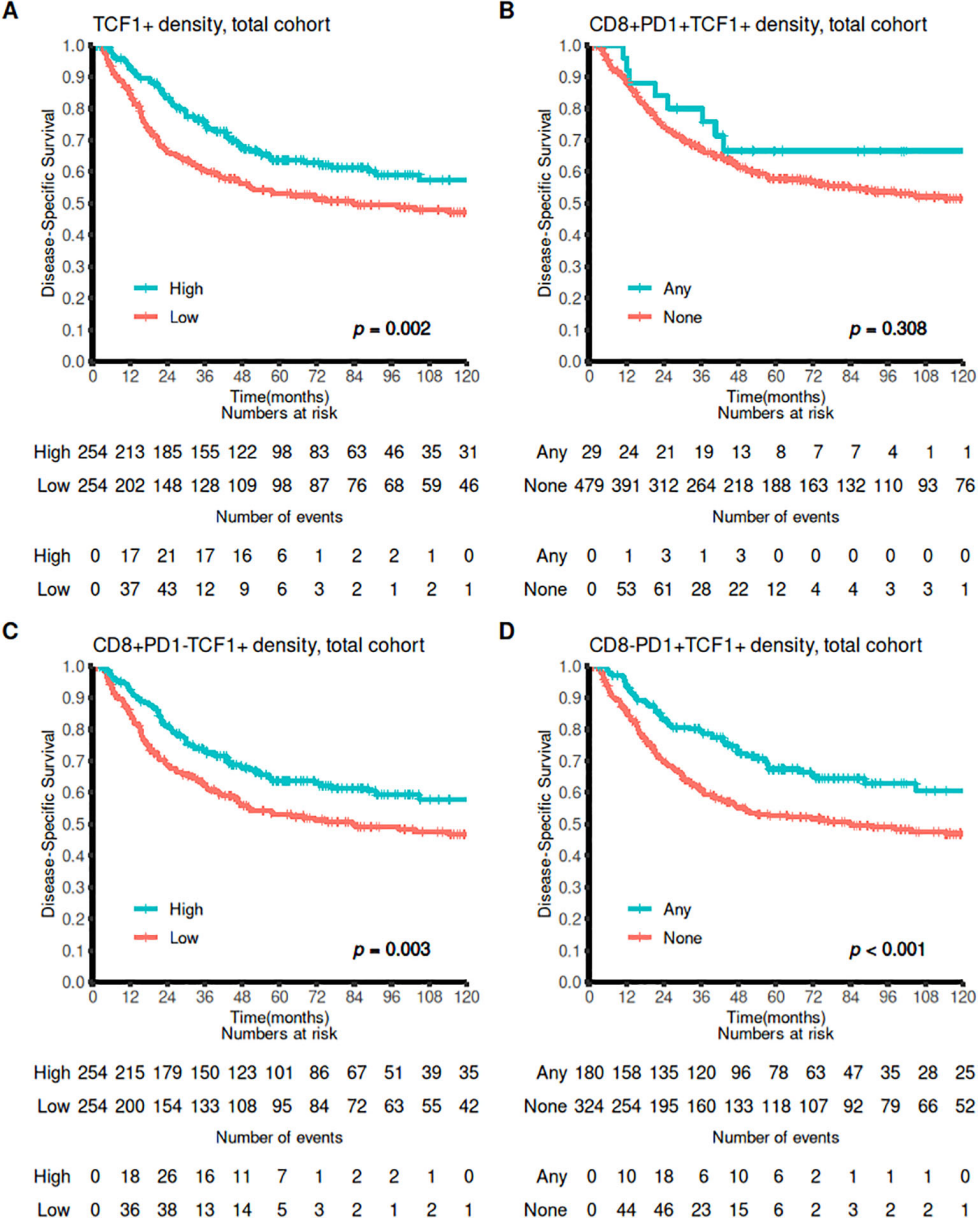
Survival curves for CD8+, PD1+ and TCF1+ markers. Association of **(A)** high vs low TCF1+ density, **(B)** Any versus none CD8+PD1+TCF1+ density **(C)** high vs low CD8+PD1-TCF1+ density, and **(D)** any versus none CD8-PD1+TCF1+ DSS outcomes presented using Kaplan-Meier curves (log-rank test).

In the whole cohort, patients with high density of TCF1 cells in tissue exhibited significantly improved DSS (p=0.002). Univariable survival analyses are found in **Tables 1**, **2** and **Figure 4**. When investigating CD8+PD1+TCF1+ cells, we found that these were present in only 29 patients. Although there was a slight trend toward improved survival in patients with triple positive cells, this difference was not statistically significant (p=0.308). However, patients with a high density of either CD8+PD1-TFC1+ or CD8-PD1+TCF1+ cells demonstrated significantly increased DSS (p-values of 0.003 and <0.001, respectively).

Multivariable survival analyses are presented in **Table 3**. These models include significant demographic and clinicopathological variables from univariable analyses: ECOG-status, histological subtype, vascular infiltration, N-, and T-status. In multivariable model 1, density of TCF1 immune cells nearly reaches significance as an independent predictor for increased DSS (HR: 0.754 95% CI: 0.0565-1.008 P=0.056). A high level of cells expressing both TCF1 and PD1 is an independent predictor for improved DSS both alone (HR: 0.612 95% CI: 0.448-0.837 P: 0.002, model 3) and together with CD8+ (HR=0.668 95% CI=0.485-0.921 P=0.014, model 2 and 4).

**Table 3:** Multivariable models.

	Model 1: Sum TCF1		Model 2: CD8+ PD1- TCF1+		Model 3: CD8- PD1+ TCF1+		Model 4: CD8- PD1+ TCF1+ vs. SumCD8	
Factor	HR (95% CI)	P	HR (95% CI)	P	HR (95% CI)	P	HR (95% CI)	p
**ECOG**		**0.016**		**0.010**		**0.026**		**0.021**
0	1 (ref)		1 (ref)		1 (ref)		1 (ref)	
1	1.53 (1.14-2.04)	**0.004**	1.56 (1.16-2.08)	**0.003**	1.49 (1.12-2.00)	**0.007**	1.51 (1.13-2.02)	**0.001**
2	1.40 (0.77-2.57)	0.273	1.46 (0.80-2.69)	0.220	1.30 (0.71-2.37)	0.399	1.34 (0.73-2.46)	0.343
**T-stage**		**<0.001**		**0.001**		**<0.001**		**0.002**
T1	1 (ref)		1 (ref)		1 (ref)		1 (ref)	
T2	1.43 (1.00-2.05)	0.053	1.45 (1.01-2.08)	**0.043**	1.41 (0.98-2.02)	0.064	1.44 (1.00-2.07)	**0.047**
T3	1.27 (0.82-1.98)	0.292	1.27 (0.81-1.97)	0.299	1.30 (0.84-2.02)	0.246	1.35 (0.87-2.10)	0.187
T4	2.52 (1.60-3.97)	**<0.001**	2.50 (1.59-3.93)	**<0.001**	2.55 (1.62-4.02)	**<0.001**	1.47 (1.57-3.89)	**<0.001**
**N-stage**		**<0.001**		**<0.001**		**<0.001**		**<0.001**
N0	1 (ref)		1 (ref)		1 (ref)		1 (ref)	
N+	2.58 (1.94-3.44)		2.58 (1.94-3.44)		2.67 (2.01-3.55)		2.68 (2.02-3.57)	
**Histology**		**0.020**		**0.035**		**0.016**		**0.018**
LUSC	1 (ref)		1 (ref)		1 (ref)		1 (ref)	
LUAD	1.49 (1.13-1.98)	**0.005**	1.45 (1.09-1.91)	**0.010**	1.51 (1.14-2.00)	**<0.004**	1.50 (1.13-1.99)	**0.005**
Other	1.03 (0.25-4.22)	0.963	1.00 (0.25-4.10)	0.997	1.04 (0.26-4.25)	0.955	1.14 (0.28-4.65)	0.857
**Vascular Infiltration**				**<0.001**		**<0.001**		**<0.001**
No	1 (ref)		1 (ref)		1 (ref)		1 (ref)	
Yes	1.83 (1.31-2.55)	**<0.001**	1.87 (1.35-2.61)		1.84 (1.32-2.56)		1.82 (1.30-2.53)	
**SumTCF1**		0.056	NE		NE		NE	
Low	1 (ref)							
High	0.75 (0.57-1.01)							
**CD8+** PD1- **TCF1+**	NE			**0.029**	NE		NE	
Low			1 (ref)					
High			0.73 (0.55-0.97)					
CD8- **TCF1+ PD1+**	NE		NE			**<0.002**		**0.014**
None					1 (ref)		1 (ref)	
Any					0.61 (0.45-0.84)		0.67 (0.49-0.92)	
**Sum CD8**	NE		NE		NE			**0.012**
Low							1 (ref)	
High							0.69(0.52-0.92)	

ECOG, Eastern Cooperative Oncology Croup Performance Status Scale; T-stage, Tumor stage; N-stage, Node stage; P-stage, Pathological stage; LUSC, Lung Squamous Cell Carcinoma; LUAD, Lung adenocarcinoma; NE, Not entered.

Backward conditional Cox-regression was performed with probabilities for stepwise entry and removal set to 0.05 and 0.10, respectively. Clinicopathological variables of significant prognostic value from the univariable analyses were entered into all multivariable analyses for DSS. P-values < 0.05 were considered statistically significant for all analyses. Median density values or any/none were used as cut-off values. Each column represents a different model using one or two cell subsets. Model 1 includes sum TCF1 (all variations of TCF1+ cell subsets), model 2 CD8+PD1-TCF1+, model 3 CD8-PD1+TCF1+. In model 4, both CD8-PD1+TCF1+ cell subsets and CD8+ total subsets were included.

Bold text indicates p-value <0.05. Shaded area visualizes variables not entered.

## Discussion

We aimed to investigate if CD8+, PD1+ and TCF1+ tumor-infiltrating lymphocytes have prognostic value in NSCLC patients. We found that CD8+PD1+TCF1+ cells were scarce and with no significant prognostic association with DSS. However, our analysis confirmed the significant prognostic value of CTLs and revealed that a high density of TCF1+ cells, co-expressed with either CD8 or PD1 are independent prognostic markers of DSS. While the impact of CD8+ cells is well documented (30, 31), the prognostic value of cells expressing TCF1+ or the TCF1+PD1+ combinations are warranted. This is the largest study in NSCLC to investigate the co-expression of CD8, PD1 and TCF1 using machine learning and digital scoring. We believe that the digital automated method to identify expression and thereby density, offers greater objectivity and reproducibility compared to the semi-quantitative methods commonly used previously by us and others (32–35).

Interestingly, patients with high density of TCF1+ tumor-infiltrating immune cells demonstrated increased DSS (p=0.002 and p=0.056 for univariable and multivariable analyses, respectively). Recent studies investigating TCF1 expressing cells in LUAD have reported similar results to ours (36–38). In a smaller immunofluorescence study, increased infiltration of CD8+TCF1+ cells were associated to improved disease-free survival and overall survival (OS). Additionally, they reported higher CD8+TCF1+ cell infiltration in lymph nodes than in primary tumors (36). Jiang et al. found that TCF+ cells were associated to recurrence-free survival (RFS) and OS. However, their subgroup analysis indicated that the best RFS and OS outcomes were linked to TCF+PD1- cells, contrasting with our results, where TCF+PD1+ cells were most significant to DSS. TCF1 was identified as a favorable prognostic marker in stage I LUAD, with significant difference in stem-like CD8 cell frequency between stage I patients and stage III patients (38). Despite some variations in the results, all three studies highlight the potential of TCF1 as a prognostic biomarker, although the impact of specific co-expression subtype varied (37).

This biomarker is of increasing interest because of its recognized function in T-cell differentiation and association with ICI efficacy. TCF1 is recognized for its regulatory role in T-cell development, important for T lineage specification, β-selection, and survival (39–42), but is also involved in the development of innate lymphoid cells (43) and NK cells (44). As previously noted, TCF1+ cells (proposed as Tpex) reside in secondary lymphoid tissues and TLS while the exhausted TCF1 low/negative cells (Tex) are observed in peripheral tissues, where antigens and infected cells are abundant (12, 18). This may suggest that formation and maintenance of TCF1+ progenitor population occurs in environments with minimal or absent antigen presence (18), supporting the hypothesis that antigen persistence contributes to the formation of Tex. The activity and function of TCF1 seems to be context dependent with the ability to influence specific gene programs within T-cells directly or indirectly, depending on the epigenetic landscape and regulatory network within each specific cell at any given time (45).

TCF1 has been demonstrated to play a key role in regulation of regulatory T cells (Tregs). A high number of Tregs is associated with poor survival in different cancer types (46), including NSCLC (47). Mammadli et al. demonstrated that the loss of TCF1 in mature T cells was found to increase Treg numbers, suggesting that TCF1 deficiency enhances Tregs suppressive functions. The authors concluded that TCF1 is important for limiting Tregs activity and their role in suppressing T cell proliferation and cytotoxicity (48). In colorectal cancer researchers observed that tumor-infiltrating Tregs had reduced TCF1 expression, along with increased Th17 and IL-17 signaling, which are proinflammatory and have tumor-promoting properties (49). This reduction in TCF1 expression in Tregs could contribute to explain our observation of improved survival in patients with a high density of TCF1+ cells as they may facilitate a more effective anti-tumor immune response. Additionally, scRNA mapping demonstrated that TCF1 represses CXRC6 expression which is abundant in chronically activated dysfunctional T cells. However, CXCR6 is also important in anti-tumor immunity, highlighting a complex relationship between TCF1, CXCR6 and T cell function (50).

Although TCF1+ cells are typically more abundant in lymphoid tissue, we and others find TCF1+ cells in NSCLC tissue (21, 23). The growing interest in TCF1+ cells mostly stem from its potential as a clinical biomarker for predicting response to immunotherapy. Many cancer patients receiving ICI treatment develop ICI resistance (17), underscoring the need to identify additional tools to predict and improve outcomes. After ICI therapy, the TCF1+and PD1+ TILs expand and generate both TCF1+ and TCF1- cells. TCF1 is required for self-renewal and stem-cell properties in CD8+ T-cells which is important for tumor control in response to ICI (16, 51). Notably, several studies have associated a high number of TCF1+ cells in NSCLC patient samples with a favorable response to ICI therapy. Higher frequencies of CD8+, PD1+ and TCF1+ cells have been observed ICI therapy responders (23). Similarly, increased numbers of PD1+TCF1+ TILS correlate with improved progression-free survival (PFS) and OS in ICI therapy responders (21). In addition, the absence of CD8+ TCF1+ T cells contributed to poor ICI responses in *STK11*-mutated NSCLC in mice (52). Higher baseline TCF1 cell levels in blood samples have been associated with clinical benefits from ICI (53). While not specific to NSCLC, Magen et al. identified an association between the number of CD8+ PD1+ TCF1+ cells in patients with a favorable response to ICI therapy in hepatocellular carcinoma (54), suggesting a broader applicability across cancer types. However, the results on TCF1+ are ambiguous as some studies reported that TCF1**-** cell populations, rather than TCF1+, favored patient survival in head and neck squamous cell cancer (55), and in preclinical models in liver cells, specific TCF1+ cells did not respond to ICI treatment (56). Despite conflicting results, most studies indicate a positive impact of TCF1 on the effectiveness of immunotherapy and survival.

Although our group have extensive experience regarding immunohistochemistry and our cohort is large, unselected and well described with a long follow-up, our study has limitations. Even after extensive training our classifier had some difficulty detecting PD1+cells, while the opposite was true for detection of TCF1+ cells, potentially leading to some false negatives and positives, respectively. Nevertheless, we believe that this potential bias is consistently spread across all analyzed cores, minimizing its impact on the results.

Compared to previous publications, our study detected a lower density of CD8+PD1+TCF1+ cells. For example, Fang et al. investigated tissue from 20 advanced NSCLC patients where the triple positive cells were identified in most biopsies, with densities ranging from 0 to 86% (23). However, Koh et al. divided their cohort of 116 ICI treated patients into patients with low or high PD1+ TCF1+ expression and stated that a considerable proportion of these patients had few if any double positive cells (21). The difference may be attributed to TMA cores versus using WSIs; particularly since TCF1 positive cells in tumor tissues are predominantly located in TLSs within stroma (12). While our TMA cores have been sampled to include both tumor and stroma, the inclusion of TLSs, if present, is coincidental. Moreover, our impression is that between TMAs and WSIs, TCF1 positive cells are generally more abundant in WSIs due to their focal localization in relation to TLSs and may not be encompassed in the small tissue area encompassed by 2-4 TMA cores present on TMA slides. Moreover, variations in stage and pretreatment between studies probably influence the descriptive results, as there is reason to believe that Tpex are more abundant in advanced and pretreated cases that are mostly represented in previous cohorts.

We were intrigued by the strong prognostic impact of CD8- PD1+/- TCF1+cells. Although triple staining with CD4, TCF1 and PD1 markers for all the TMA slides was outside the scope of this study, we still wanted to explore which subset of cells these CD8-TCF1+ represented. Our combined findings suggest that CD4+ TCF1+PD1+/- cells may have prognostic significance in NSCLC. CD4+ T cells are crucial for supporting or suppressing cytotoxic CD8+ T cell responses, and TCF1 is important for CD4 T cell stemness (57, 58). The prognostic relevance of this subtype is yet to be explored and can only be suggested by our findings, highlighting a key area for future research.

It is important to highlight that although TCF1+ cells are predominantly lymphoid, TCF1 expression is also present in NK cells (59). Based on the limited WSI data, we estimate that around 2% of TCF1+ cells were neither CD8+ or CD4+, suggesting the potential presence of NK cells. However, NK cells are typically few in tumor tissue (60). As previously mentioned, this observation is limited to a single patient slide. Furthermore, correlation analysis with CD56, a well-known NK cell surface marker, revealed no significant correlation with TCF1 expression. This implies that NK cells do not make a substantial contribution to the TCF1+ cell population in NSCLC tissue.

In this study, we utilized an objective and easily reproducible scoring method to investigate the presence of CD8+, PD1+ and TCF1+ cells in a large cohort of surgically resected NSCLC patients not otherwise treated, revealing a low occurrence of triple positive cells in our cohort. Other researchers have found that presence of these cells is of particular importance in patients receiving immunotherapy, and that these cells increase in proliferation after ICI treatment (21, 23, 51), highlighting their possible predictive potential. While we found no significant prognostic impact of triple positive cells, we identified TCF1 as a potential prognostic biomarker for DSS in NSCLC, at least when co-expressed with CD8 or PD1. However, we must interpret with caution when using TMA for studying these focally located cells. Future research should aim to investigate TCF1 further in whole slides, exploring the spatial distribution of these cells by IHC or immunofluorescence. To better understand the underlying mechanisms of the immune responses in NSCLC, there is also a need to further investigate the role of the CD4+TCF1+ subtype, which is probably more abundant and potentially have a prognostic impact, suggested by our exploration. Our study contributes to the understanding of NSCLC immune cell infiltration, the strong prognostic impact of various subtypes of TCF1+ cells and could aid in future development of personalized immunotherapy strategies based on the immune environment in NSCLC.

## Data Availability

The raw data supporting the conclusions of this article will be made available by the authors, without undue reservation.

## References

[1] Ferlay J Colombet M Soerjomataram I Parkin DM Piñeros M Znaor A. Cancer statistics for the year 2020: An overview. Int J Cancer. (2021) 149:778–89. doi: 10.1002/ijc.v149.4 33818764

[2] Kreftregisteret. Årsrapport Nasjonalt kvalitetsregister for lungekreft 2023. Oslo: Cancer Registry of Norway (2023). Available at: https://www.kreftregisteret.no/Registrene/Kvalitetsregistrene/Kvalitetsregister-for-lungekreft (accessed May 15, 2024).

[3] Bray F Laversanne M Sung H Ferlay J Siegel RL Soerjomataram I. Global cancer statistics 2022: GLOBOCAN estimates of incidence and mortality worldwide for 36 cancers in 185 countries. CA Cancer J Clin. (2024) 74:229–63. doi: 10.3322/caac.21834 38572751

[4] Duma N Santana-Davila R Molina JR. Non-small cell lung cancer: epidemiology, screening, diagnosis, and treatment. Mayo Clin Proc. (2019) 94:1623–40. doi: 10.1016/j.mayocp.2019.01.013 31378236

[5] Gonzalez H Hagerling C Werb Z. Roles of the immune system in cancer: from tumor initiation to metastatic progression. Genes Dev. (2018) 32:1267–84. doi: 10.1101/gad.314617.118 PMC6169832 30275043

[6] Hanahan D Weinberg RA. Hallmarks of cancer: the next generation. Cell. (2011) 144:646–74. doi: 10.1016/j.cell.2011.02.013 21376230

[7] Raskov H Orhan A Christensen JP Gögenur I. Cytotoxic CD8+ T cells in cancer and cancer immunotherapy. Br J Cancer. (2021) 124:359–67. doi: 10.1038/s41416-020-01048-4 PMC7853123 32929195

[8] Farhood B Najafi M Mortezaee K. CD8(+) cytotoxic T lymphocytes in cancer immunotherapy: A review. J Cell Physiol. (2019) 234:8509–21. doi: 10.1002/jcp.v234.6 30520029

[9] Yi L Yang L. Stem-like T cells and niches: Implications in human health and disease. Front Immunol. (2022) 13. doi: 10.3389/fimmu.2022.907172 PMC9428355 36059484

[10] Philip M Schietinger A. CD8(+) T cell differentiation and dysfunction in cancer. Nat Rev Immunol. (2022) 22:209–23. doi: 10.1038/s41577-021-00574-3 PMC9792152 34253904

[11] McLane LM Abdel-Hakeem MS Wherry EJ. CD8 T cell exhaustion during chronic viral infection and cancer. Annu Rev Immunol. (2019) 37:457–95. doi: 10.1146/annurev-immunol-041015-055318 30676822

[12] Im SJ Obeng RC Nasti TH McManus D Kamphorst AO Gunisetty S. Characteristics and anatomic location of PD-1(+)TCF1(+) stem-like CD8 T cells in chronic viral infection and cancer. Proc Natl Acad Sci U S A. (2023) 120:e2221985120. doi: 10.1073/pnas.2221985120 37782797 PMC10576122

[13] Utzschneider DT Charmoy M Chennupati V Pousse L Ferreira DP Calderon-Copete S. T cell factor 1-expressing memory-like CD8(+) T cells sustain the immune response to chronic viral infections. Immunity. (2016) 45:415–27. doi: 10.1016/j.immuni.2016.07.021 27533016

[14] Zhang J Lyu T Cao Y Feng H. Role of TCF-1 in differentiation, exhaustion, and memory of CD8(+) T cells: A review. FASEB J. (2021) 35:e21549. doi: 10.1096/fj.202002566R 33913198

[15] Gounari F Khazaie K. TCF-1: a maverick in T cell development and function. Nat Immunol. (2022) 23:671–8. doi: 10.1038/s41590-022-01194-2 PMC9202512 35487986

[16] Zhao X Shan Q Xue H-H. TCF1 in T cell immunity: a broadened frontier. Nat Rev Immunol. (2022) 22:147–57. doi: 10.1038/s41577-021-00563-6 34127847

[17] Jung S Baek JH. The potential of T cell factor 1 in sustaining CD8(+) T lymphocyte-directed anti-tumor immunity. Cancers (Basel). (2021) 13(3):515. doi: 10.3390/cancers13030515 33572793 PMC7866257

[18] Philip M Schietinger A. Heterogeneity and fate choice: T cell exhaustion in cancer and chronic infections. Curr Opin Immunol. (2019) 58:98–103. doi: 10.1016/j.coi.2019.04.014 31181510 PMC7608527

[19] Wieland D Kemming J Schuch A Emmerich F Knolle P Neumann-Haefelin C. TCF1+ hepatitis C virus-specific CD8+ T cells are maintained after cessation of chronic antigen stimulation. Nat Commun. (2017) 8:15050. doi: 10.1038/ncomms15050 28466857 PMC5418623

[20] Im SJ Hashimoto M Gerner MY Lee J Kissick HT Burger MC. Defining CD8+ T cells that provide the proliferative burst after PD-1 therapy. Nature. (2016) 537:417–21. doi: 10.1038/nature19330 PMC5297183 27501248

[21] Koh J Kim S Woo YD Song SG Yim J Han B. TCF1(+)PD-1(+) tumour-infiltrating lymphocytes predict a favorable response and prolonged survival after immune checkpoint inhibitor therapy for non-small-cell lung cancer. Eur J Cancer. (2022) 174:10–20. doi: 10.1016/j.ejca.2022.07.004 35970031

[22] Chen JH Nieman LT Spurrell M Jorgji V Elmelech L Richieri P. Human lung cancer harbors spatially organized stem-immunity hubs associated with response to immunotherapy. Nat Immunol. (2024) 25:644–58. doi: 10.1038/s41590-024-01792-2 PMC12096941 38503922

[23] Fang X Wu G Hua J Zhao P Shan M Wang N. TCF-1(+) PD-1(+) CD8(+)T cells are associated with the response to PD-1 blockade in non-small cell lung cancer patients. J Cancer Res Clin Oncol. (2022) 148:2653–60. doi: 10.1007/s00432-021-03845-7 PMC11800887 34725738

[24] Rakaee M Busund LR Jamaly S Paulsen EE Richardsen E Andersen S. Prognostic value of macrophage phenotypes in resectable non-small cell lung cancer assessed by multiplex immunohistochemistry. Neoplasia. (2019) 21:282–93. doi: 10.1016/j.neo.2019.01.005 PMC6369140 30743162

[25] Hald SM Rakaee M Martinez I Richardsen E Al-Saad S Paulsen EE. LAG-3 in non-small-cell lung cancer: expression in primary tumors and metastatic lymph nodes is associated with improved survival. Clin Lung Cancer. (2018) 19:249–59.e2. doi: 10.1016/j.cllc.2017.12.001 29396238

[26] Bremnes RM Veve R Gabrielson E Hirsch FR Baron A Bemis L. High-throughput tissue microarray analysis used to evaluate biology and prognostic significance of the E-cadherin pathway in non-small-cell lung cancer. J Clin Oncol. (2002) 20:2417–28. doi: 10.1200/JCO.2002.08.159 12011119

[27] Donnem T Kilvaer TK Andersen S Richardsen E Paulsen EE Hald SM. Strategies for clinical implementation of TNM-Immunoscore in resected nonsmall-cell lung cancer. Ann Oncol. (2016) 27:225–32. doi: 10.1093/annonc/mdv560 26578726

[28] Bankhead P Loughrey MB Fernández JA Dombrowski Y McArt DG Dunne PD. QuPath: Open source software for digital pathology image analysis. Sci Rep. (2017) 7:16878. doi: 10.1038/s41598-017-17204-5 29203879 PMC5715110

[29] Schmidt U Weigert M Broaddus C Myers G. (2018)., in: Cell Detection with Star-Convex Polygons: 21st International Conference, Granada, Spain, September 16–20, 2018. pp. 265–73, Proceedings, Part II.

[30] Hald SM Bremnes RM Al-Shibli K Al-Saad S Andersen S Stenvold H. CD4/CD8 co-expression shows independent prognostic impact in resected non-small cell lung cancer patients treated with adjuvant radiotherapy. Lung Cancer. (2013) 80:209–15. doi: 10.1016/j.lungcan.2012.12.026 23384671

[31] Donnem T Hald SM Paulsen EE Richardsen E Al-Saad S Kilvaer TK. Stromal CD8+ T-cell density—A promising supplement to TNM staging in non-small cell lung cancer. Clin Cancer Res. (2015) 21:2635–43. doi: 10.1158/1078-0432.CCR-14-1905 25680376

[32] Rakaee M Busund LT Paulsen EE Richardsen E Al-Saad S Andersen S. Prognostic effect of intratumoral neutrophils across histological subtypes of non-small cell lung cancer. Oncotarget. (2016) 7:72184–96. doi: 10.18632/oncotarget.12360 PMC5342153 27708229

[33] Rakaee M Kilvaer TK Dalen SM Richardsen E Paulsen EE Hald SM. Evaluation of tumor-infiltrating lymphocytes using routine H&E slides predicts patient survival in resected non-small cell lung cancer. Hum Pathol. (2018) 79:188–98. doi: 10.1016/j.humpath.2018.05.017 29885403

[34] Brambilla E Le Teuff G Marguet S Lantuejoul S Dunant A Graziano S. Prognostic effect of tumor lymphocytic infiltration in resectable non-small-cell lung cancer. J Clin Oncol. (2016) 34:1223–30. doi: 10.1200/JCO.2015.63.0970 PMC4872323 26834066

[35] Ruffini E Asioli S Filosso PL Lyberis P Bruna MC Macrì L. Clinical significance of tumor-infiltrating lymphocytes in lung neoplasms. Ann Thorac Surgery. (2009) 87:365–72. doi: 10.1016/j.athoracsur.2008.10.067 19161739

[36] Wang Y Ma L Chen Y Yun W Yu J Meng X. Prognostic effect of TCF1+ CD8+ T cell and TOX+ CD8+ T cell infiltration in lung adenocarcinoma. Cancer Sci. (2024) 115:2184–95. doi: 10.1111/cas.v115.7 PMC11247562 38590234

[37] Jiang K Liu S Chen Y Xu Z Xu Z Qian B. Characterization of TCF-1 and its relationship between CD8+ TIL densities and immune checkpoints and their joint influences on prognoses of lung adenocarcinoma patients. Thorac Cancer. (2023) 14:2745–53. doi: 10.1111/1759-7714.15058 PMC10518226 37536668

[38] Sang J Liu P Wang M Xu F Ma J Wei Z. Stem-like CD8 T cells in stage I lung adenocarcinoma as a prognostic biomarker: A preliminary study. J Cancer Res Ther. (2024) 20:669–77. doi: 10.4103/jcrt.jcrt_2453_23 38687939

[39] Ioannidis V Beermann F Clevers H Held W. The beta-catenin–TCF-1 pathway ensures CD4(+)CD8(+) thymocyte survival. Nat Immunol. (2001) 2:691–7. doi: 10.1038/90623 11477404

[40] Germar K Dose M Konstantinou T Zhang J Wang H Lobry C. T-cell factor 1 is a gatekeeper for T-cell specification in response to Notch signaling. Proc Natl Acad Sci U S A. (2011) 108:20060–5. doi: 10.1073/pnas.1110230108 PMC3250146 22109558

[41] Weber BN Chi AW Chavez A Yashiro-Ohtani Y Yang Q Shestova O. A critical role for TCF-1 in T-lineage specification and differentiation. Nature. (2011) 476:63–8. doi: 10.1038/nature10279 PMC3156435 21814277

[42] Gullicksrud JA Shan Q Xue H-H. Tcf1 at the crossroads of CD4+ and CD8+ T cell identity. Front Biol. (2017) 12:83–93. doi: 10.1007/s11515-017-1445-3

[43] Raghu D Xue HH Mielke LA. Control of lymphocyte fate, infection, and tumor immunity by TCF-1. Trends Immunol. (2019) 40:1149–62. doi: 10.1016/j.it.2019.10.006 31734149

[44] Liu J Wang Z Hao S Wang F Yao Y Zhang Y. Tcf1 sustains the expression of multiple regulators in promoting early natural killer cell development. Front Immunol. (2021) 12:791220. doi: 10.3389/fimmu.2021.791220 34917097 PMC8669559

[45] Escobar G Mangani D Anderson AC. T cell factor 1: A master regulator of the T cell response in disease. Sci Immunol. (2020) 5(53). doi: 10.1126/sciimmunol.abb9726 PMC8221367 33158974

[46] Ohue Y Nishikawa H. Regulatory T (Treg) cells in cancer: Can Treg cells be a new therapeutic target? Cancer Sci. (2019) 110:2080–9. doi: 10.1111/cas.2019.110.issue-7 PMC6609813 31102428

[47] Duan MC Zhong XN Liu GN Wei JR. The Treg/Th17 paradigm in lung cancer. J Immunol Res. (2014) 2014:730380. doi: 10.1155/2014/730380 24872958 PMC4020459

[48] Mammadli M Suo L Sen JM Karimi M. TCF-1 negatively regulates the suppressive ability of canonical and noncanonical Tregs. J Leukoc Biol. (2023) 113:489–503. doi: 10.1093/jleuko/qiad019 36806938 PMC11651127

[49] Osman A Yan B Li Y Pavelko KD Quandt J Saadalla A. TCF-1 controls T(reg) cell functions that regulate inflammation, CD8(+) T cell cytotoxicity and severity of colon cancer. Nat Immunol. (2021) 22:1152–62. doi: 10.1038/s41590-021-00987-1 PMC8428683 34385712

[50] Tooley K Jerby L Escobar G Krovi SH Mangani D Dandekar G. Pan-cancer mapping of single CD8+ T cell profiles reveals a TCF1:CXCR6 axis regulating CD28 co-stimulation and anti-tumor immunity. Cell Rep Med. (2024) 5:101640. doi: 10.1016/j.xcrm.2024.101640 38959885 PMC11293343

[51] Siddiqui I Schaeuble K Chennupati V Fuertes Marraco SA Calderon-Copete S Pais Ferreira D. Intratumoral tcf1(+)PD-1(+)CD8(+) T cells with stem-like properties promote tumor control in response to vaccination and checkpoint blockade immunotherapy. Immunity. (2019) 50:195–211.e10. doi: 10.1016/j.immuni.2018.12.021 30635237

[52] Li H Liu Z Liu L Zhang H Han C Girard L. AXL targeting restores PD-1 blockade sensitivity of STK11/LKB1 mutant NSCLC through expansion of TCF1(+) CD8 T cells. Cell Rep Med. (2022) 3:100554. doi: 10.1016/j.xcrm.2022.100554 35492873 PMC9040166

[53] Maniar R Wang PH Washburn RS Kratchmarov R Coley SM Saqi A. Self-renewing CD8+ T-cell abundance in blood associates with response to immunotherapy. Cancer Immunol Res. (2023) 11:164–70. doi: 10.1158/2326-6066.CIR-22-0524 PMC9898128 36512052

[54] Magen A Hamon P Fiaschi N Soong BY Park MD Mattiuz R. Intratumoral dendritic cell-CD4(+) T helper cell niches enable CD8(+) T cell differentiation following PD-1 blockade in hepatocellular carcinoma. Nat Med. (2023) 29:1389–99. doi: 10.1038/s41591-023-02345-0 PMC11027932 37322116

[55] Wang D Fang J Wen S Li Q Wang J Yang L. A comprehensive profile of TCF1(+) progenitor and TCF1(-) terminally exhausted PD-1(+)CD8(+) T cells in head and neck squamous cell carcinoma: implications for prognosis and immunotherapy. Int J Oral Sci. (2022) 14:8. doi: 10.1038/s41368-022-00160-w 35153298 PMC8841504

[56] Roetman JJ Erwin MM Rudloff MW Favret NR Detrés Román CR Apostolova MKI. Tumor-reactive CD8+ T cells enter a TCF1+PD-1- dysfunctional state. Cancer Immunol Res. (2023) 11:1630–41. doi: 10.1158/2326-6066.CIR-22-0939 PMC10841346 37844197

[57] Mammadli M Suo L Sen JM Karimi M. TCF-1 is required for CD4 T cell persistence functions during alloImmunity. Int J Mol Sci. (2023) 24(5):4326. doi: 10.3390/ijms24054326 36901757 PMC10002223

[58] Nish SA Zens KD Kratchmarov R Lin WW Adams WC Chen YH. CD4+ T cell effector commitment coupled to self-renewal by asymmetric cell divisions. J Exp Med. (2017) 214:39–47. doi: 10.1084/jem.20161046 27923906 PMC5206501

[59] Jeevan-Raj B Gehrig J Charmoy M Chennupati V Grandclément C Angelino P. The transcription factor tcf1 contributes to normal NK cell development and function by limiting the expression of granzymes. Cell Rep. (2017) 20:613–26. doi: 10.1016/j.celrep.2017.06.071 28723565

[60] Portale F Di Mitri D. NK cells in cancer: mechanisms of dysfunction and therapeutic potential. Int J Mol Sci. (2023) 24:9521. doi: 10.3390/ijms24119521 37298470 PMC10253405

[61] McShane LM Altman DG Sauerbrei W Taube SE Gion M Clark GM. REporting recommendations for tumor MARKer prognostic studies (REMARK). Nat Clin Pract Oncol. (2005) 2:416–22. doi: 10.1038/ncponc0252 16130938

