# Supplementary material for: High density of TCF1+ stem-like tumor-infiltrating lymphocytes is associated with favorable disease-specific survival in NSCLC
Source: Front Immunol. 2024 Dec 19;15:1504220. doi: 10.3389/fimmu.2024.1504220 (PMC11693705; doi:10.3389/fimmu.2024.1504220)
Supplement: Supplementary file 1 [file Table1.docx]

# Supplementary CD8PD1TCF


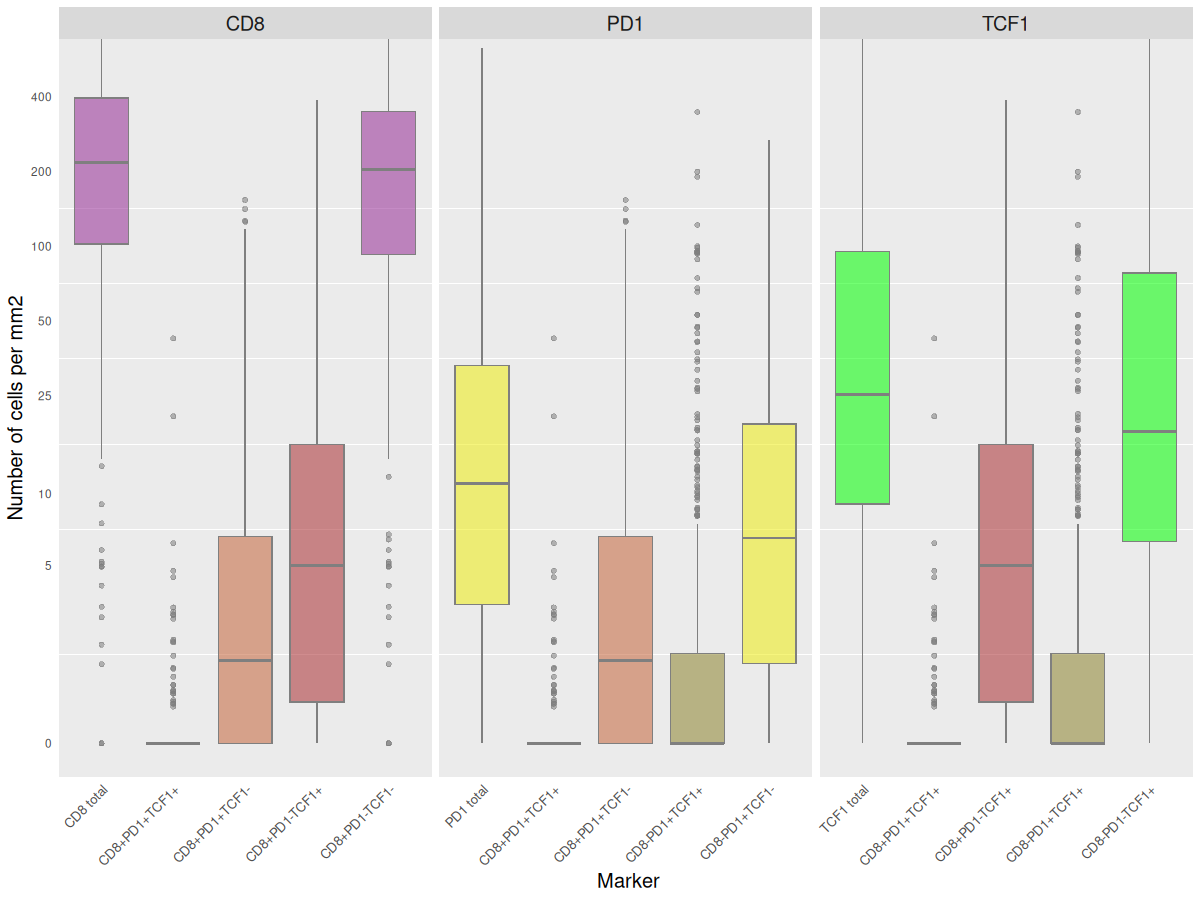


**Figure S1:** Immune cells densities on TMA-slides. Shown is the total number of CD8, PD1 and TCF1 positive cells and their different subtypes per mm^2^. Log scale. The mean number of CD8+/TCF1+ cell were 19.83/mm2, while TCF1+/PD1+ cells measured 6.22 cells/mm2.

**Immunhistochemistry protocol description**

IHC triple stain was performed in Discovery Ultra Research instrument, Roche 05987750001. Formalin-fixed, paraffin-embedded 4-μm-thick TMA and whole lung tissue sections were used for this study. We used validated antibodies for IHC-P from Cell Signaling and IVD approved antibodies for in vitro diagnostic use from Ventana Roche/Sigma Aldrich. *In house,* each antibody was optimized as a single stain before setting up together in sequences. In triple stain order of antibodies and chromogens were tested for best quality regarding localization of antigen and expression of chromogens. Controls from whole lung cancer tissue and a multi tissue TMA control of positive and negative tissues were used during optimization and final runs.

Green, Purple and Yellow chromogen detection kits visualized the localization of each

marker. Green detection showed a clear green color in nucleus expression for TCF1. Purple and Yellow gave membranous staining for CD4 and CD8. In addition, Yellow was mainly cytoplasmatic expressed for PD1. Purple and Yellow detections are translucent chromogens and will give a third red/orange color if antigens are co-localized near each other in the same cell.

Protocol in short

All sections were baked, deparaffinized and retrieved before three sequences of antibody detection. Between sequences a stripping step with denaturation and neutralization was done to avoid cross reactions of antibodies and enzymes. Blocking of unspecific bindings and signal amplifications, were used when required. Primary antibody and secondary multimer antibodies were incubated before final chromogen detection. Blue counterstain in nucleus was done by hematoxylin. After detection sections were dehydrated and mounted.

| **Tissue**  **Baking** | **Depar.** | **Antigen**  **Retrieval** | **Block.** | **Primary Ab** | **Block.** | **Secondary**  **Multimer** | **Amplify** | **Detection** | **Counter**  **stain** |
| --- | --- | --- | --- | --- | --- | --- | --- | --- | --- |
| SEQUENCE 1 | | | | | | | | | |
| 8 min  60^o^C | Disco. wash  3x12 min  68^o^C | CC1  40 min  95^o^C | Inhib 12 min  37^o^C | Ab 1  Rb mono  TCF-1  1:100  60 min  36^o^C | - | OMap  anti-Rb HRP  16 min  37^o^C | AMP  TSA HQ  8/8 min  37^o^C | Green HRP  32/16min  37^o^C | - |
| Stripping step: Denatruration CC2, 8 min, 100 C^o^ Neutralize H_2_O_2_ Inhibitor, 20 min, 40^o^C | | | | | | | | | |
| SEQUENCE 2 | | | | | | | | | |
| - | - | - | - | Ab 2  Rb mono  CD8  Predil.  32 min  36^o^C | AB blokk  16 min | UMap  anti-Rb  HRP  16 min  37^o^C | - | Purple/  H_2_O_2_  24 min  37^o^C |  |
| Stripping step: Denatruration CC2, 8 min, 100 C^o^ Neutralize H_2_O_2_ Inhibitor, 20 min, 40^o^C | | | | | | | | | |
| SEQUENCE 3 | | | | | | | | | |
| - | - | - | - | Ab 3  Ms mono  PD1  Predil.  60 min  36^o^C | AB blokk  16 min | UMap  anti-Ms  AP  32 min  37^o^C | Anti-HQ AP  32 min | Yellow AP  H_2_O_2_  2 Hr 40 min  37^o^C | Hem II/  Bluing  4/4min  37^o^C |

Table S1. IHC Triple stain Protocol for CD8, PD1, TCF1 TMA

| **Tissue**  **Baking** | **Depar.** | **Antigen**  **Retrieval** | **Block.** | **Primary Ab** | **Block.** | **Secondary**  **Multimer** | **Amplify** | **Detection** | **Counter**  **stain** |
| --- | --- | --- | --- | --- | --- | --- | --- | --- | --- |
| SEQUENCE 1 | | | | | | | | | |
| 8 min  60^o^C | Disco. wash  3x12 min  68^o^C | CC1  40 min  95^o^C | Inhib 12 min  37^o^C | Ab 1  Rb mono  TCF-1  1:100  60 min  36^o^C | - | OMap  anti-Rb HRP  16 min  37^o^C | AMP  TSA HQ  8/8 min  37^o^C | Green HRP  32/16min  37^o^C | - |
| Stripping step: Denatruration CC2, 8 min, 100 C^o^ Neutralize H_2_O_2_ Inhibitor, 20 min, 40^o^C | | | | | | | | | |
| SEQUENCE 2 | | | | | | | | | |
| - | - | - | - | Ab 2  Rb mono  CD4  Predil.  32 min  36^o^C | AB blokk  16 min | UMap  anti-Rb  HRP  16 min  37^o^C | - | Purple/  H_2_O_2_  12 min  37^o^C |  |
| Stripping step: Denatruration CC2, 8 min, 100 C^o^ Neutralize H_2_O_2_ Inhibitor, 20 min, 40^o^C | | | | | | | | | |
| SEQUENCE 3 | | | | | | | | | |
| - | - | - | - | Ab 3  Rb mono  CD8  Predil.  32 min  36^o^C |  | OMap  anti-Rb  HRP  16 min  37^o^C | - | Yellow HRP  H_2_O_2_  48/40 min  37^o^C | Hem II/  Bluing  4/4min  37^o^C |

Table S2. IHC Triple stain Protocol for CD8, CD4 and TCF1 WSI

Table S3. Product references for immunohistochemistry

| **Antibodies and Reagents** | **Reference** | **Company** |
| --- | --- | --- |
| CONFIRM anti-CD8 (SP7) | 05937248001 | Roche |
| CONFIRM anti- CD4 (SP35) | 05552737001 | Roche |
| PD-1 (NAT105) | 07099029001 | Roche/Sigma-Aldrich |
| TCF1/TCF7 (C63D9) | #2203 | Cell Signaling |
| Discovery Wash | 7311079001 | Roche |
| Ultra LCS (Liquid cover slip) | 5424534001 | Roche |
| Reaction buffer (10x) | 5353955001 | Roche |
| Discovery CC1 RUO | 6414575001 | Roche |
| Antibody Dilution buffer | 5280524001 | Roche |
| Benchmark Ultra CC2 | 5424542001 | Roche |
| Discovery Inhibitor RUO | 7017944001 | Roche |
| Discovery Antibody block RUO | 5268869001 | Roche |
| Discovery AMP HQ kit RUO | 6472320001 | Roche |
| Discovery anti HQ HRP RUO | 6442544001 | Roche |
| Anti HQ AP | 6472311001 | Roche |
| OmniMap anti-Ms HRP RUO | 5269652001 | Roche |
| OmniMap anti-Rb HRP RUO | 5269679001 | Roche |
| UMap anti-Rb HRP | 5269717001 | Roche |
| UMap anti-Ms HRP | 5269695001 | Roche |
| Discovery Green HRP kit RUO | 8478295001 | Roche |
| Discovery Purple Kit RUO | 7053983001 | Roche |
| Yellow AP | 7698445001 | Roche |
| Yellow HRP | 8502641001 | Roche |
| Hematoxylin II | 5277965001 | Roche |
| Bluing Reagent | 5266769001 | Roche |
| Ethanol 96% | 20823.362 | VWR, Avantor |
| Etnanol absolute | 20821.296 | VWR, Avantor |
| Xylene | 28975.291 | VWR, Avantor |
| Histokitt mounting medium | Assistant 1025/250 | Sondheim/Rhoen |
